# Seasonal insights for integrative mosquito management from multi-year baseline entomological data on *Aedes aegypti* in Lee County, Florida

**DOI:** 10.1371/journal.pone.0311407

**Published:** 2024-10-11

**Authors:** Rachel Morreale, Steven Stenhouse, Danilo O. Carvalho, Daniel A. Hahn, Kostas Bourtzis, Aaron Lloyd, Thomas Wayne Gale, David F. Hoel

**Affiliations:** 1 Lee County Mosquito Control District, Lehigh Acres, FL, United States of America; 2 Insect Pest Control Subprogramme, Joint FAO/IAEA Centre of Nuclear Techniques in Food and Agriculture, IAEA, Vienna, Austria; 3 Department of Entomology & Nematology, University of Florida, Gainesville, FL, United States of America; Clinton Health Access Initiative, UNITED STATES OF AMERICA

## Abstract

The spread of arboviruses like yellow fever, dengue, chikungunya, and Zika, transmitted by the invasive mosquito *Aedes aegypti* has led to the development of many strategies to suppress mosquito populations. Given the rapid development of resistance to common chemical larvicides and adulticides in some *Ae*. *aegypti* populations, as well as the ever-shrinking chemical options for mosquito control, there is a pressing need for new tools and deployment of those innovative tools as a component of integrative mosquito management programs. Prior to the adoption of any mosquito population intervention, be it conventional or innovative, understanding the baseline population is essential to evaluate the efficacy of the control measure. The Lee County Mosquito Control District in Florida has collected a three-year-long period of baseline entomological surveillance data collection for *Ae*. *aegypti* on Captiva and Sanibel Islands as foundational information prior to implementation of a new integrative mosquito management approach. We identified 18 mosquito species and described their population dynamics during the rainy and dry seasons. The two islands had no significant differences in species richness, diversity, dominance, or evenness overall. Yet, there were clear differences between the high rain season and low rain season in the Shannon diversity index, Simpson dominance index, and Pielou species evenness index within each site. Our data suggest that any innovative intervention should begin before mid to late April when the mosquito population is at its lowest and certainly before populations build up to their summer peak between June and September. These data also show the spatial distribution of *Ae*. *aegypti* is dynamic in space and time, identifying hotspots of mosquito abundance to focus on for future interventions. Overall, our study emphasizes the importance of entomological data collection to understand the population dynamics of *Ae*. *aegypti* mosquitoes, including the impact of environmental factors such as temperature and precipitation.

## 1. Introduction

Arboviruses, such as yellow fever, dengue, chikungunya, and Zika, have burdened the public health system in many tropical and subtropical countries [1]. These diseases are transmitted by mosquitoes during blood feeding, in which the principal vector is the female of *Aedes aegypti* (L.) [2]. This species is invasive in tropical and sub-tropical locales world-wide and flourishes in urban environments, showing incredible resilience [2]. It can be found in a broad range of breeding sites and environments worldwide, needing only small amounts of standing water with low organic matter for development [2]. Suppressing mosquito populations can disrupt the disease cycle, reducing the infection risk. However, current methods of population suppression often center around the application of insecticides, which can have unintentional effects on non-target species and the environment as well as the selection of pesticide resistance [3–6]. Furthermore, most ultralow-volume spray treatments have low success in reaching this species’ cryptic breeding and resting sites. As a result, the impact of conventional chemical insecticides on *Ae*. *aegypti* control is low [7].

Alternative methods to chemical pesticides to suppress *Ae*. *aegypti* populations, thereby reducing the chances of disease transmission, are now under evaluation in several countries [8–10]. Autocidal techniques, where mosquitoes of the same species are used to kill other mosquitoes, are growing in their potential for effectively suppressing *Ae*. *aegypti* and *Ae*. *albopictus* populations in the wild and are also being explored for other essential vector species. An example of such a control tactic is the sterile insect technique (SIT), which is based on the release of large numbers of sterile male mosquitoes of the target species in the field to increase the chances of a wild female mating with an infertile male instead of mating with a (fertile) wild male. Consecutive releases of sterile males can increase the chances of sterile male mating and cause population collapse over the generations by transferring sterile sperm to wild females, thus introducing sterility to the target population [11]. SIT has been successfully deployed in the field to control populations of *Ae*. *aegypti* and *Ae*. *albopictus* [12–15]. Similarly, the incompatible insect technique (IIT) uses the bacteria *Wolbachia* to induce reproductive incompatibility between wild females and lab-reared males infected with a strain of *Wolbachia* not present in the wild females [9]. IIT is much like SIT in deployment and operations, except that *Wolbachia*-based cytoplasmic incompatibility is used to prevent wild female reproduction rather than the double-stranded DNA damage that induces sterility in SIT [9], and IIT has also successfully been used to suppress *Ae*. *aegypti* and *Ae*. *albopictus* in operational field trials [16–18]. Evaluation of the efficacy of autocidal techniques requires substantial baseline knowledge about the target wild mosquito populations [9, 19, 20]. This information includes the sizes and spatial distributions of wild populations so control efforts can release appropriate numbers of lab-reared mosquitoes to successfully impact the wild population and deploy those releases at sites within the intervention area with the greatest densities of wild mosquitoes. Similarly, all of these autocidal techniques work best if they begin when wild populations are at their lowest. For example, it is often recommended that SIT or IIT releases begin during the early spring before wild populations have grown substantially from their low overwintering densities so that population suppression can be enacted with fewer lab-reared mosquitoes released [11, 21]. Thus, precise estimates of seasonality in wild population abundances are needed to time the early releases of lab-reared mosquitoes in autocidal programs to achieve maximum potential suppression of wild mosquitoes in the field.

Overall, we believe that careful population monitoring is beneficial before applying any innovative intervention. For example, in the sterile insect technique (SIT), to achieve operational and large-scale production for field applications, SIT projects should begin as a pilot trial based on a phased conditional approach that starts with preintervention baseline data collection, small-scale field trials, preoperational, and operational phases [19]. Baseline data collection is essential for determining the mosquito population profile by deploying traps for different life stages (usually adults and eggs) in the target and control areas. The objective is to collect data to characterize the target mosquito species’ population profile and other species that may be attracted to the same traps. More broadly, autocidal techniques are species-specific and do not alter populations of non-target species present in the target area, making the collection of non-target species a critical component of successfully showing that the intervention affected only the target species without substantially affecting other co-occurring species [11]. Data on abiotic parameters, such as temperature and precipitation, that can influence the mosquito population profile, potentially predicting their peaks, spatial distribution, and size, should also be collected during baseline population monitoring [22–25]. Collection of baseline data for an extended period is crucial as it allows for comparisons among time points (i.e., years) and seasons, thus providing solid evidence for the validation of population suppression or elimination in the target area [11].

Regardless of the control method (conventional or innovative), there is a need for robust field data collection. The American Mosquito Control Association (AMCA) listed the main advantages of surveillance as 1) “*requesting appropriate resources as part of a needs assessment;* 2) *determining changes in the geographic distribution and abundance of mosquito species;* 3) *evaluating control efforts by comparing pre-and post-surveillance data;* 4) *obtaining relative measurements of the vector populations over time and accumulating a historical database;* 5) *facilitating appropriate and timely decisions regarding interventions”* [26]. The AMCA reinforces that entomological surveillance provides the opportunity to compare historical data to current data, helping mosquito control professionals make operational decisions and determine when action thresholds have been met, according to local and national regulations [26]. In addition, the European Mosquito Control Association (EMCA) emphasizes the importance of tracking invasive species capable of transmitting diseases and autochthonous species local to that area as a preventive initiative to avoid disseminating vector-borne diseases [27–29].

To enhance the management of the invasive, disease-vectoring mosquito *Ae*. *aegypti*, The Lee County Mosquito Control District (LCMCD) in Florida (USA) has completed a 3-year baseline entomological surveillance study on Captiva Island (intervention area) and Sanibel Island (non-intervention area). From mid-2017 until mid-2020 we collected mosquitoes from a network of 58 trapping sites, each with BG-Sentinel traps and ovitraps, spread across our focal areas on the two islands to describe the seasonal abundance, diversity, and distributions of *Ae*. *aegypti* and the allied mosquito community in our field sites over 3 years. These data will provide essential information for conventional chemical control applications and innovative control techniques for controlling *Aedes aegypti* that will ultimately improve LCMCD’s current integrative mosquito control program. While these data have many possible uses, LCMCD is especially focused on their application for an operational sterile insect technique (SIT) program evaluation targeting *Ae*. *aegypti*, and we interpret many of our observations through this lens.

## 2. Material and methods

### 2.1. Ethical considerations

The creation, existence, and activities of the Mosquito Control Districts in Florida are regulated under the 2018 Florida Statutes, title XXIX (Public Health), chapter 388 (Mosquito Control), in sections 171 and 181, which state the “*Power to perform work*” and the “*Power to do all things necessary*”, in which states “*The respective districts of the state are hereby fully authorized to do and perform all things necessary to carry out the intent and purposes of this law*”. With that said, there was no further authorization necessary to develop and apply mosquito trapping within the County.

### 2.2. Study area

Captiva Island was selected as the intervention area for the suppression of the *Ae*. *aegypti*. Areas of beaches and mangrove swamps were omitted because they are not breeding sites for this target species. Hence, the study area covered about 230 ha of the island’s 419.6 ha of total surface area. An area of 38 ha in the northwest part of Sanibel Island, connected to the southern part of Captiva Island by an automotive bridge, was selected for the non-intervention area as preliminary data indicated that it has similar environmental and presence of *Ae*. *aegypti* density conditions, allowing systematic and comprehensive monitoring.

### 2.3. Weather data

Temperature and precipitation data were obtained from the nearest available weather stations from the National Weather Service from the National Oceanic and Atmospheric Administration (NOAA) from mid-2017 until mid-2020. Because there were no closer weather stations collecting both parameters, the weather station that recorded precipitation was located in St. James City, Pine Island, FL (26.494623, -82.077372, around 12 km away), while temperature was recorded from a weather station in Fort Myers, FL (26.58495, -81.86146, around 34 km away). Precipitation data were divided between low and high rainy seasons; the high rainy season typically lasts from the second week of May until the second week of October, when precipitation events occur several times a week with regularity, and we consider the rest of the year the low rainy season when precipitation is sporadic.

### 2.4. Mosquito trapping

**Fig 1:**
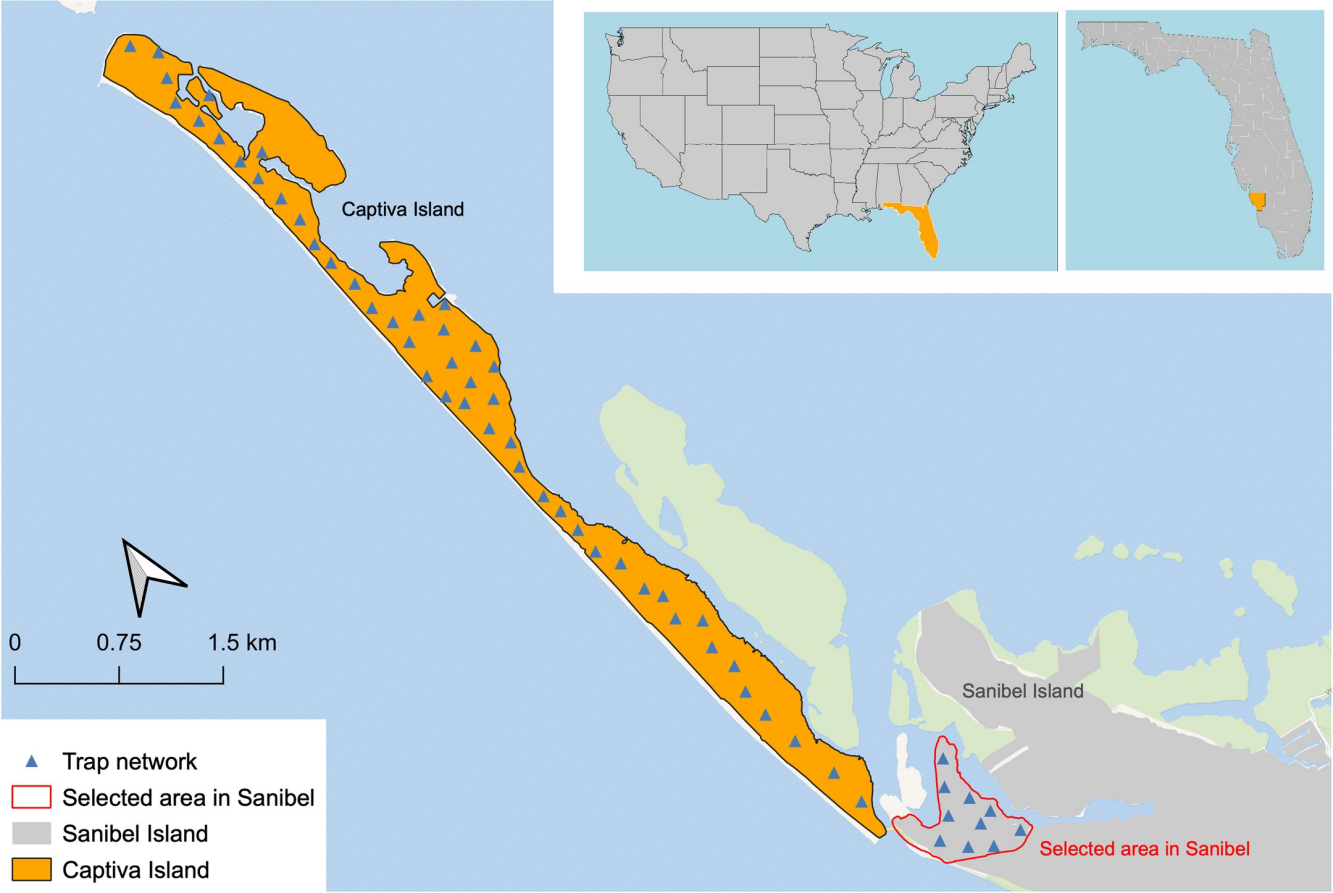
Study area. Map of Captiva and northwest Sanibel Islands in Lee County (FL, USA) SIT intervention (orange) and non-intervention (outlined in red) areas, including GPS positioning of the trapping stations (represented with a triangle) deployed for the collection of baseline entomological data from mid-2017 to mid-2020.

Surveillance began in June 2017, with 58 trapping stations distributed across both areas (Fig 1). These locations were selected based on previous trapping studies in which the presence of *Ae*. *aegypti* populations had been documented. Trap sites were selected based on the appropriate habitat and easy accessibility to the location, with a preference for areas that were unobtrusive to residents and were less likely to be interfered with. Surveillance points were placed approximately every 200 m throughout Captiva with a total of 48 points in this configuration, along with ten trapping stations on Sanibel. The three trapping stations covering the southernmost 1 km of Captiva were placed every 300 m.

#### 2.4.1. Adult trapping

Adult mosquitoes (Culicidae) were collected using BG-Sentinel 2 traps (BGS) baited with a BG-Lure in all 58 trapping stations. Trapping was conducted twice per week unless canceled due to extreme weather or LCMCD closure (i.e., during holidays), reducing collection events to once per week or omitting the trapping that week. Traps were placed in the field for approximately 24 hours. Collected samples were put into a cooler with dry ice to immediately knock down collected mosquitoes to better preserve specimens for identification. Mosquitoes were identified using dichotomous keys [30, 31]. *Culex* identified in the subgenus *Melanoconion* were not identified at the species level; instead, they were grouped as *Cx*. *melanoconion* for analyses. Very few individual mosquitoes were damaged enough by collection that they could not be identified to species when brought back to the laboratory. Specimens that could not be identified to species were not included in the analysis presented here, but it was overall less than ~0.038% of the total individuals sampled.

#### 2.4.2. Egg trapping

Eggs were collected weekly using ovitraps (OVT) unless during LCMCD closure or extreme weather events in which the OVTs were collected from the field in advance. The OVT consisted of a 473 ml black plastic cup stationed near each the BGSs. A hole was drilled approximately 2 cm from the top of the cup to allow excess water that may enter the cup due to rainfall to drain out. A trifold paper towel was replaced weekly into the cups as the oviposition medium. Any remaining water in the cup was fully drained before collection. Cups were then rinsed and replenished with approximately 200 ml of water. Oviposition papers were returned to the laboratory, where they were allowed to dry under insectary conditions. Once the egg papers dried, the numbers of *Aedes* eggs were quantified based on microscope examination.

### 2.5. Statistical analysis

Ecological parameters were calculated based on common indices for describing communities: the Margalef index for species richness, the Shannon index for diversity, and the Simpson index for dominance, all obtained using the package *vegan* for R [32]. Pielou’s species evenness index was calculated by *E* = *H*/ln *S*, where *H* is the Shannon diversity index, and *S* is the number of species; the value varies between 0 and 1, where 1 represents an utterly even distribution and 0 a completely clumped distribution. The Sørensen dissimilarity index was used between Captiva and Sanibel areas, defined as *Qs* = (2 × *J*)/(*a* + *b*), where *J* represents the number of species in common in both areas, while *a + b* indicates the total number of species found in each area. The relative abundance of mosquito species across the communities in each area was classified according to the description of Trojan (1992), in which *satellite* species correspond to less than 1% of the relative abundance (RA), *sub-dominant* species RA < 5%, and *dominant* species RA > 5%. Relative abundance was calculated based on the specimens of each species over the total captured throughout both areas [33]. The association index (Ai) was defined *Ai* = 2[*c*/(*x* + *y*) − 0.5], where *c* is the number of individuals of both species in samples where they occur together, while *x* or *y* is the total number of individuals of each species in all collected samples. The spatial distribution of each species in each area was obtained using the kernel density estimation from the software QGIS (version 3.22.10). All statistical analyses were performed using R and RStudio (libraries and versions available in supplementary material). The non-metric multidimensional scaling (NMDS) ordination process was performed in the package *vegan* for R [32], building a Bray-Curtis distance matrix and targeting a stress value around 0.2. The Permutational Multivariate Analysis of Variance Using Distance Matrices was implemented to test for differences between the two areas in either the low or high rainy season. The hotspot analysis begins by defining spatial neighbors using the *dnearneigh* function, which establishes spatial relationships based on a specified distance threshold. Subsequently, the *nb2listw* function is employed to convert the neighbor object into a spatial weights matrix, crucial for quantifying the strength of spatial connections. The *localG* function is then applied to compute local Moran’s I statistics, offering a localized perspective on spatial autocorrelation. This integrated approach, leveraging *dnearneigh* for neighbor specification and *nb2listw* for spatial weights, enhances the capability to discern fine-grained spatial patterns and identify local clusters or outliers within the dataset.

## 3. Results

### 3.1. Baseline entomological data on Captiva and Sanibel Islands

Using the 58 BGSs shown in Fig 1, adult mosquito collections operated from mid-June 2017 until mid-June 2020 resulting in the identification of seven Culicidae genera and 18 species between the proposed intervention area on Captiva Island and the non-intervention area on Sanibel Island. Through BGS collections over the course of three years, a total of 75 286 and 25 302 adult mosquitoes (both males and females) were collected on Captiva and Sanibel, respectively. On Captiva the most abundant species were *Ae*. *aegypti* with 53 090 individuals (70.5%), *Culex quinquefasciatus* with 15 239 individuals (20.2%), and *Ae*. *taeniorhynchus* with 2 838 individuals (3.77%). On Sanibel *Ae*. *aegypti* was also the most abundant species with 19 781 individuals (78.2%), followed by *Ae*. *taeniorhynchus* with 1 849 individuals (7.31%) and *Cx*. *nigripalpus* with 1 204 individuals (4.76%).

Table 1 shows the relative abundance (RA) of all species collected on Captiva and Sanibel. Overall, *Ae*. *aegypti* and *Cx*. *quinquefasciatus* were the most dominant species in the BGSs collections, with relative abundances of 72.4% and 16.2%, respectively. *Ae*. *taeniorhynchus*, *Cx*. *nigripalpus*, and *Wyeomyia mitchelli* were sub-dominant, with relative abundance values of 4.66%, 3.45%, and 1.72%, respectively. All other trap-collected species were classified as satellite species with relative abundance values below 0.7%, with *Mansonia titillans*, *Psorophora ferox*, and *Aedes albopictus* having the lowest values. Reinforcing the dominance of *Ae*. *aegypti* in these locations, most species showed low association with *Ae*. *aegypti*, generating association index values close to -1 (Table 1). The only exception was that *Cx*. *quinquefasciatus* had a low positive association index value of 0.134, showing the two species co-occurred in traps occasionally.

**Table 1:** Total number of mosquitoes collected.

Genera	Species	Island		Relative Abundance		Association index
		Sanibel	Captiva			
*Aedes*	*aegypti*	19 781	53 090	72.4	Dominant	-
	*albopictus*	2	1	0.003	Satellite	-0.999
	*atlanticus*	34	18	0.052		-0.997
	*sollicitans*	-	8	0.008		-0.998
	*taeniorhynchus*	1 849	2 838	4.66	Sub-dominant	-0.472
	*triseriatus*	49	185	0.233	Satellite	-0.906
	*vexans*	-	1	0.0001		-0.999
*Anopheles*	*atropos*	15	299	0.312		-0.976
	*crucians*	12	31	0.043		-0.996
	*quadrimaculatus*	7	6	0.013		-0.996
*Culex*	*melanoconion*	145	501	0.642		-0.939
	*nigripalpus*	1 204	2 270	3.45	Sub-dominant	-0.690
	*quinquefasciatus*	1 060	15 239	16.2	Dominant	0.134
*Mansonia*	*titillans*	-	1	0.0001	Satellite	-1.000
*Psorophora*	*columbiae*	52	95	0.146		-0.959
	*ferox*	1	1	0.002		-0.999
*Uranotaeni*	*lowii*	12	51	0.063		-0.992
*Wyeomyia*	*mitchellii*	1 079	651	1.72	Sub-dominant	-0.765

Total numbers of adult mosquitoes collected using BGSs in Captiva and Sanibel from mid-2017 to mid-2020 by species, accompanied by their abundances and associations. Relative abundance was classified in both areas as follows: Satellite (RA < 1%), sub-dominant (RA < 5%), and dominant (RA > 5%). Association index was calculated for Ae. aegypti. Dashed missing values mean that the species was not found in that area.

Using these data, a series of ecological indexes were calculated for Sanibel and Captiva Islands during the high and low-rain seasons as a reference for future interventions (Table 2). Throughout the study period, there was no difference between the sites on Sanibel and Captiva in the species richness index (3.86 and 3.49 for Sanibel and Captiva, respectively, t-test = 0.82, df = 12.74, *p* = 0.43). The Shannon diversity index was estimated at 0.98 and 0.93 for Sanibel and Captiva, respectively, with no statistical difference between the two sites detected across the three years (t-test = 0.56, df = 23.17, *p* = 0.58). The dominance index reached 0.46 and 0.48 for Sanibel and Captiva, respectively, but did not differ between the two areas (t-test = 0.43, df = 23.23, *p* = 0.66). Furthermore, the evenness ratio, which is the relative abundance of different species in the same area (defined as the species’ evenness), was similar between the two sites (0.47 and 0.48 for Sanibel and Captiva respectively, t = 0.28, df = 27.92, *p* = 0.781). Comparing the high and low-rainy seasons in both places, the similarity quotient indicated that the sites were similar irrespective of the season, 1.12 and 1.07 for the high and low-rain seasons, with an overall similarity quotient throughout the years equal to 1.11.

**Table 2:** Ecological indexes.

Indexes	Areas	Sanibel			Captiva		
	Rainy season	High	Low	Total	High	Low	Total
Mean number of species		9.40	9.00	8.70	8.17	6.81	7.59
Richness*		1.40	1.46	1.38	1.46	1.45	1.51
Diversity		0.77	1.19	0.98	0.85	1.00	0.93
Dominance		0.36	0.57	0.46	0.42	0.55	0.49
Species Evenness**		0.34	0.54	0.47	0.42	0.54	0.48
Quotient of similarity***		-	-	-	1.12	1.07	1.10

Ecological indices for the study areas during the high and low rainy seasons from mid-2017 to mid-2020 including the

* Margalef index for richness

** the Pielou index for species evenness, and the

*** Sørensen dissimilarity index that was calculated with the correspondent Sanibel area as the reference.

**Fig 2:**
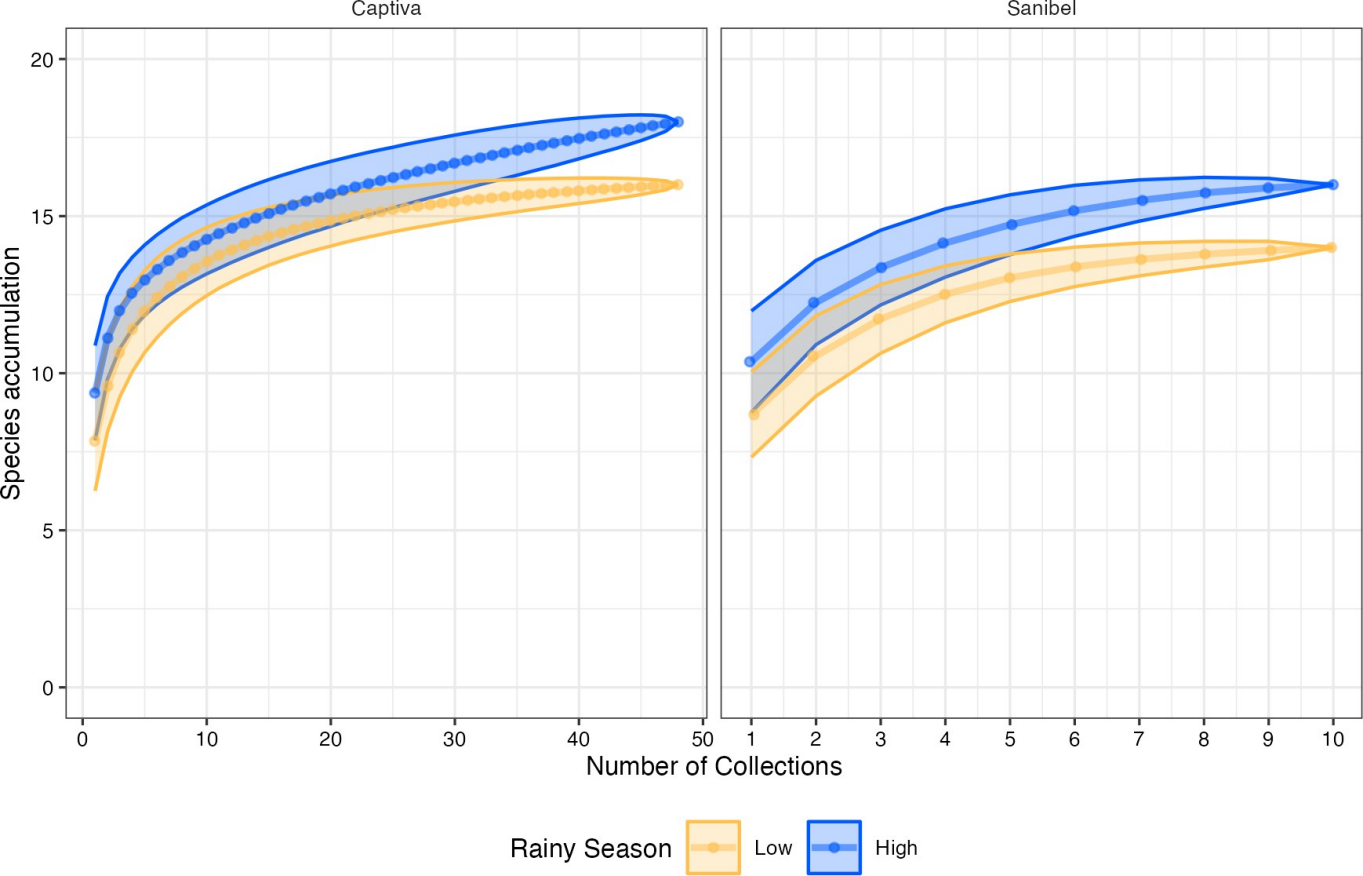
Species accumulation. Species accumulation curve for Captiva and Sanibel Islands during the low (orange) and high (blue) rainy season as sampling size or number of recaptures increases.

Although the mean number of species on Captiva trended towards being lower in the low-rain season compared to the high-rain season, 6.81 and 8.17 respectively, there was no statistically significant difference in the mean number of species trapped between the two seasons (t-test = -0.69, df = 92.4, *p* = 0.49). Fig 2 shows the species accumulation curve based on the number of samples and traps, where the cumulative number of species discovered (y-axis) is plotted against the cumulative number of individuals sampled (x-axis) according to the seasonality for Captiva and Sanibel. In both cases, the high-rain season has slightly faster species accumulation over time than the low-rain season, reaching a plateau. However, the Shannon diversity index was 0.85 and 1.0 for the high and low-rain seasons, respectively, showing that species diversity was actually slightly higher in the low-rain season than the high-rain season (t-test = 22.41, df = 91.41, *p* = 0.02). The Simpson dominance index was greater in the low-rain season than in the high-rain season (0.49 vs. 0.42 t-test = 4.4, df = 85.2, *p* = 3.18^−5^), demonstrating that *Ae*. *aegypti* was relatively more prevalent than any other species during the low-rain season than the high-rain season even if the absolute numbers of *Ae*. *aegypti* captured were substantially lower in the low-rain season. Similarly, the evenness of species across the areas sampled was greater in the low-rain season that during the high-rain season, Pielou index of 0.54 vs 0.42 (t-test = 3.6, f = 91.63, *p* = 4.2^−4^). In contrast, no statistically significant difference was observed concerning the Margalef index of richness between the low and the high-rain season, 1.40 and 1.46, respectively (t-test = 0.63, df = 17.65, *p* = 0.53).

**Fig 3:**
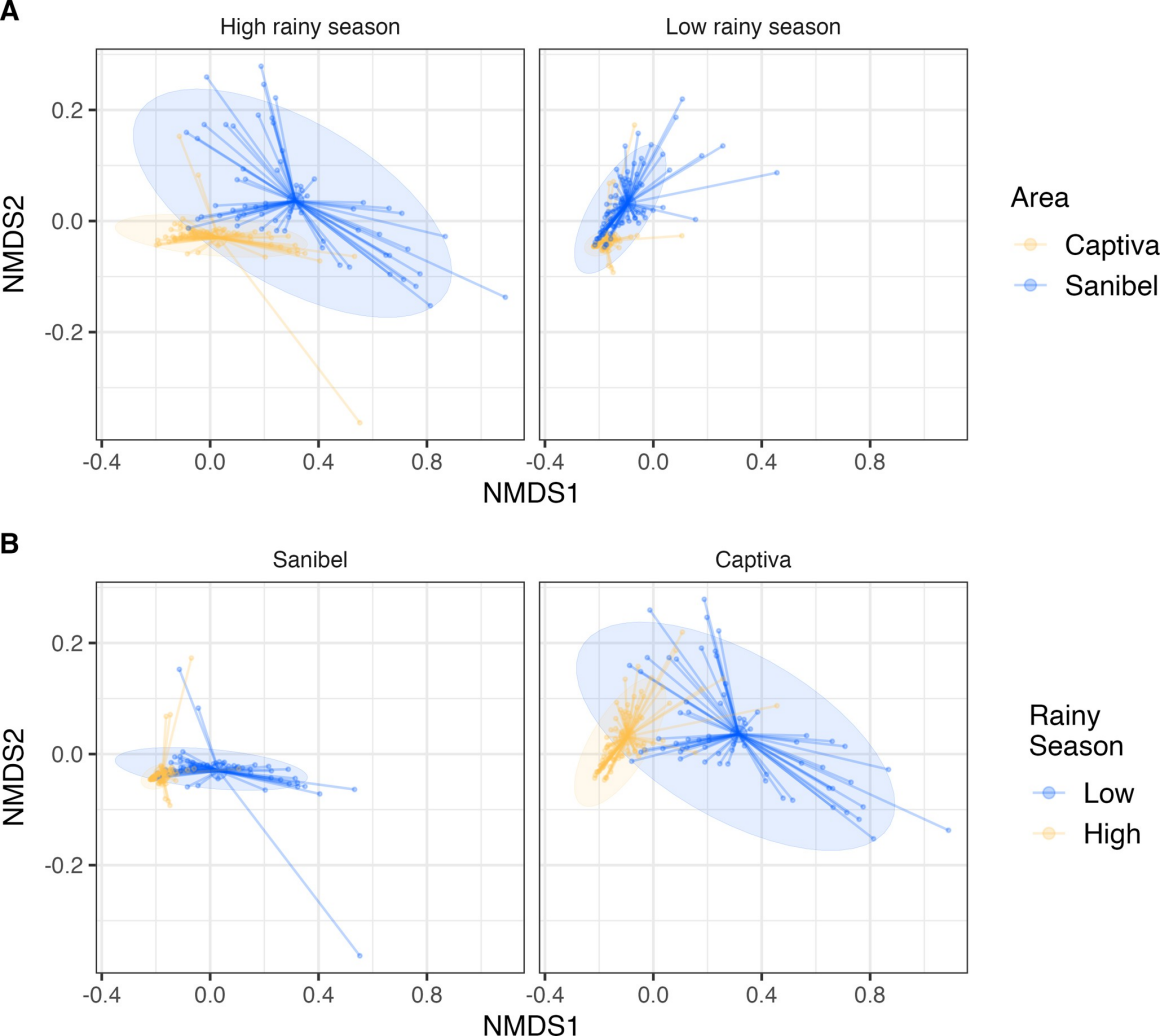
Non-metric multidimensional scaling. NMDS ordination from all mosquito species found during the low and high rainy seasons (A) in Captiva and Sanibel (B). The Bray-Curtis dissimilarity matrix was used to determine dissimilarities among mosquito community compositions as an arbitrary distance matrix—stress value = 0.05.

To compare species composition between the sites on Captiva and Sanibel we applied a non-metric multidimensional scaling (NMDS) ordination process separately in the high and low-rainy seasons (Fig 3A) and the same analysis was also used to partition the high vs. low rainy season within each site (Fig 3B). A Permutational Multivariate Analysis of Variance (PERMANOVA) using distance matrices was performed to test for effects of location, Captiva vs. Sanibel, and season, high vs. low-rain season. Reinforcing the above data showing little to no difference in species composition between Sanibel vs. Captiva, the multivariate species composition was detectably different between Sanibel vs. Captiva (p<0.001), but site only explained approximately 3.86% of the total variance in the multivariate species distance matrix among traps (Fig 3A). Perhaps more interesting was that there were substantial differences in the multivariate species composition between traps collected in the high vs. low-rainy season on both Sanibel and Captiva (p<0.001), with season explaining approximately 52.08% of the variance in the multivariate species composition among traps (Fig 3B). Despite the potential contradiction between the two indices (Shannon’s diversity and Simpson’s abundance), their high values presented spatial overlaps, in which some trapping stations had high values for diversity and dominance both on Captiva and Sanibel (S1 Fig). The contradictory intersection of the indexes may represent the overlap of several ecological niches that might be occupied by different species, contributing to those values of abundance and diversity in the trapping station area.

### 3.2. *Aedes aegypti* population dynamics

**Fig 4:**
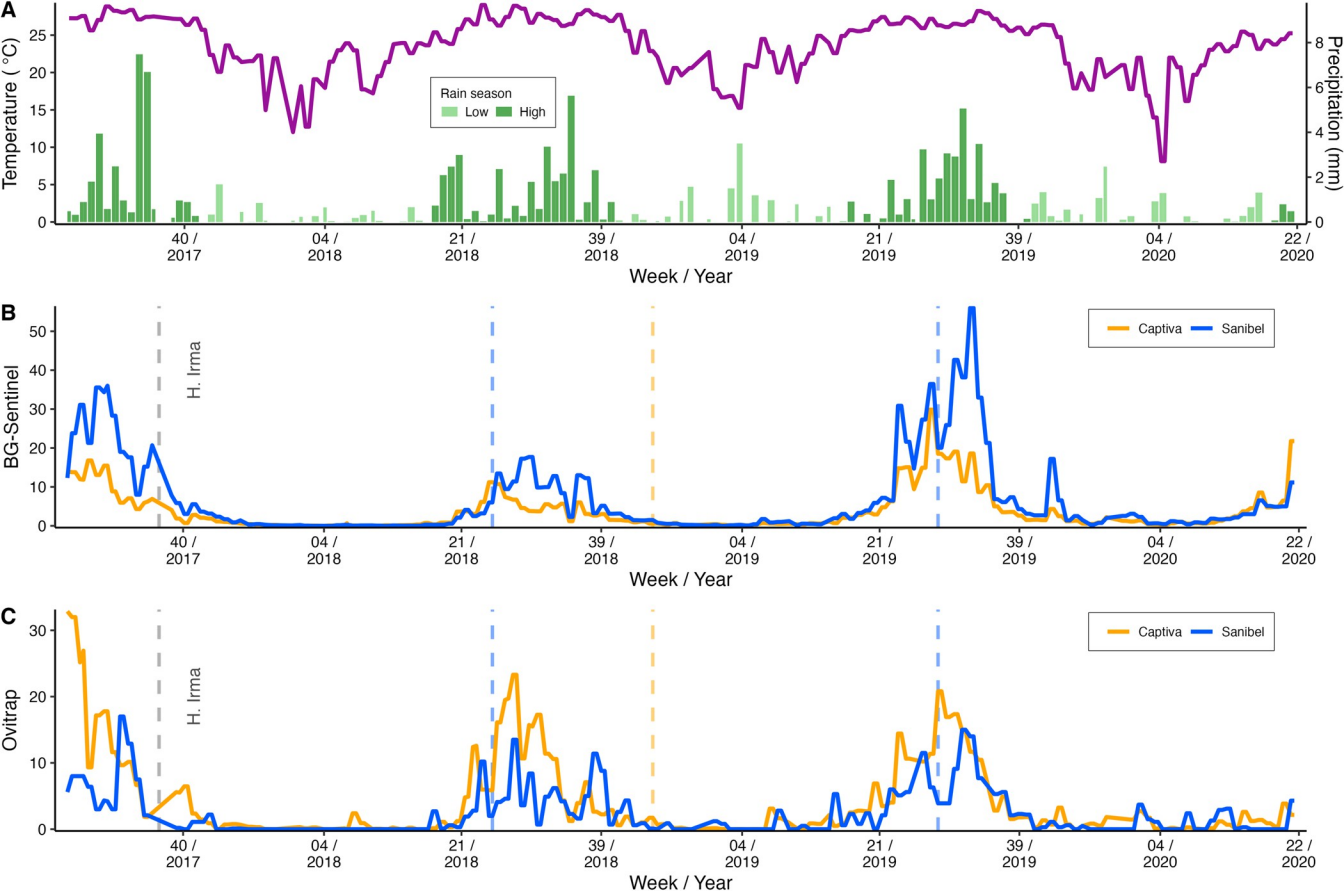
Weather and *Aedes* data profile. Weather profile with the mean weekly temperature (black line) and weekly precipitation records (green bars with dark green denoting the high-rain season and light green denoting the low-rain season) (A), the total number of adult *Ae*. *aegypti* collected in BGSs each week (B), and the number of ovitraps containing *Aedes* eggs each week (C) from the study areas–Captiva and Sanibel with their respective weekly means. Hurricane Irma hit the area on September 10^th^, 2017 (grey dashed lines). Rare collection events of *Ae*. *albopictus* in Sanibel and Captiva are also noted by blue and orange dashed lines, respectively, representing one specimen collected by the BG-Sentinel trap in the weeks denoted. *Aedes albopictus* collections happened on Sanibel during the high-rain season while on Captiva, *Ae*. *albopictus* was found during the low-rain season.

Climatic conditions were associated with predictable fluctuations in the numbers of *Ae*. *aegypti* adults collected in traps, with higher abundance during the high-rain season and lower abundance in the low-rain season across each of the three years (Fig 4A and 4B). *Aedes aegypti* trap captures were positively, correlated with weekly precipitation levels, albeit weakly (R^2^ = 0.23 with S = 8.02^11^, *p* < 0.05). There was a slightly higher positive correlation of *Ae*. *aegypti* abundance with weekly mean temperature (R^2^ = 0.54 with S = 4.94^11^, *p* < 0.05), as may be observed between panels in Fig 4. *Aedes aegypti* was dispersed throughout the intervention and non-intervention sites, with a significant decline during the low rainy season (GLM F = 129.9, df = 1, *p* < 0.05), resulting in 86.9% and 89.4% reduction of the adult population in Captiva and Sanibel, respectively, during the low-rain season. Egg collections from ovitraps had a similar pattern as adult captures (Fig 4C), in which *Ae*. *aegypti* egg density had clear seasonal fluctuations across all three years and a higher correlation with temperature (R^2^ = 0.35 with S = 8.33^10^, *p* < 0.05) compared with precipitation (R^2^ = 0.11 with S = 1.13^11^, *p* < 0.05).

Before this study, and as shown in LCMCD historical monitoring data, *Ae*. *albopictus*, a species very closely related to *Ae*. *aegypti* for arbovirus transmission, ecological niche, and blood meal host preference, had not been detected on Captiva and only rarely had been detected in routine surveillance on Sanibel Island. During the rigorous and consistent trapping of this study, however, *Ae*. *albopictus* was detected in the non-intervention area of Sanibel in week #30 in 2018 and a second time in week #26 in 2019. *Aedes albopictus* was also detected for the first time in Captiva in week #46 in 2018. In all three cases, a single individual was collected. No further *Ae*. *albopictus* collections were recorded in either area during the three-year-long study period. These highly sporadic *Ae*. *albopictus* collections could indicate separate introduction events without establishment, as we failed to consistently detect *Ae*. *albopictus* through subsequent sampling.

### 3.3. Describing the distribution of *Ae*. *aegypti* on Captiva and Sanibel

**Fig 5:**
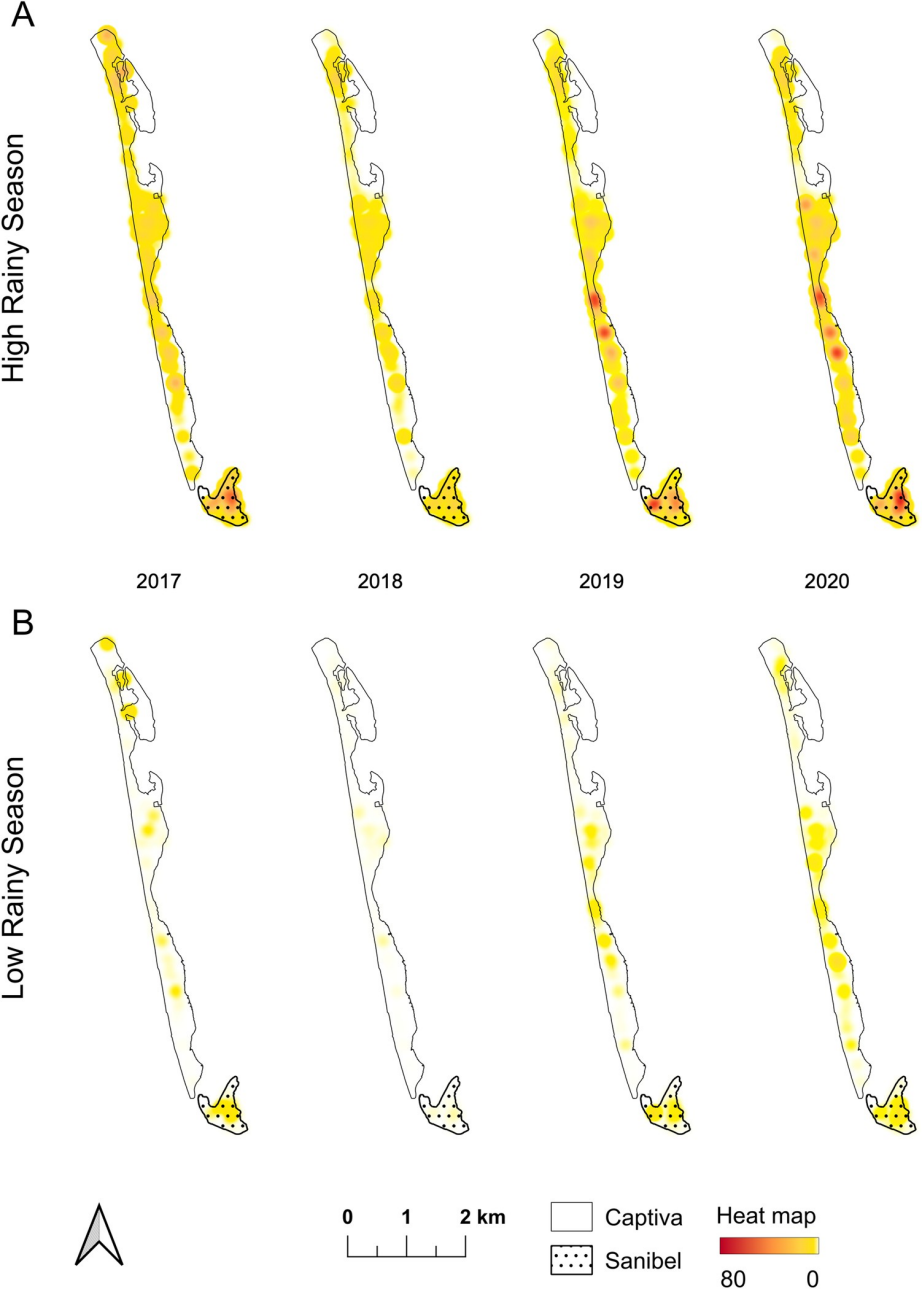
Spatial distribution of adult. Visualization of the spatial distribution of adult *Ae*. *aegypti* collected in BG-Sentinel traps during the low and high-rain seasons from mid-2017 to mid-2020 using kernel density estimation interpolation.

**Fig 6:**
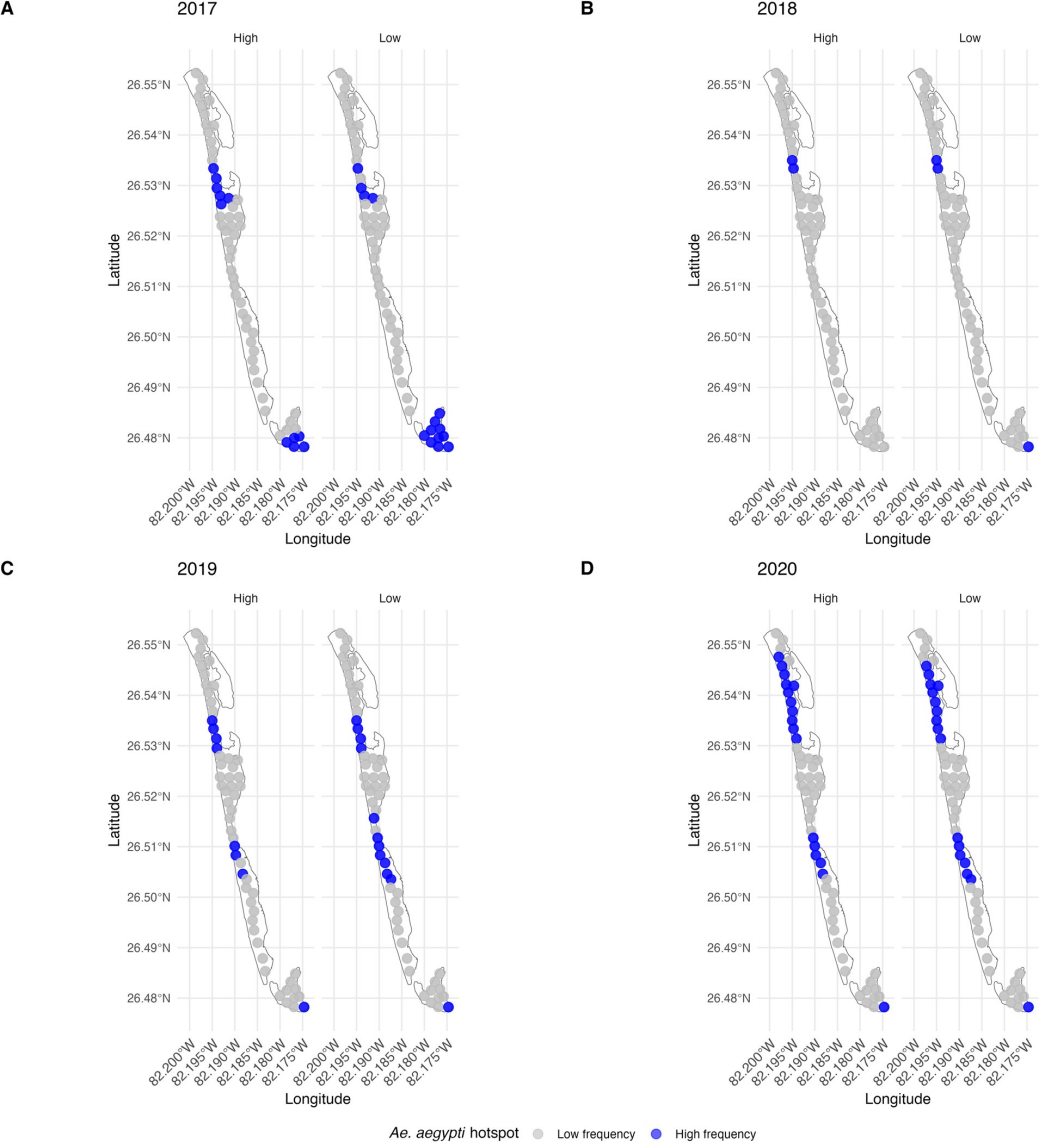
Clustering hotspot. Hotspots of adult *Ae*. *aegypti* identified by clustering of collections BG-Sentinel traps during the low and high-rain seasons from mid-2017 to mid-2020 identified by the Local Moran’s I method where traps that had repeatable significantly high captures are shown in blue and traps without repeatable high captures are shown in grey.

As a first pass towards identifying areas with greater *Ae*. *aegypti* density within our study areas, we used the kernel density extrapolation to estimate trap captures for *Aedes aegypti*, in Captiva and Sanibel during the high and low rain season to generate a heatmap for abundance by trap (Fig 5). Visual inspection of the heatmap suggested that there were potential hotspots of higher *Ae*. *aegypti* density, where we may target future interventions. Consistent with the analyses in the section above, visual inspection of the heatmaps separated by high vs. low-rain season in each year reinforces the point that *Ae*. *aegypti* populations are much lower in the low-rain season than in high-rain season. Beyond our first-pass visual inspections of the heat maps, we used the Local Moran’s I statistic to identify significant areas of spatial clustering according to trap position in the field, thereby quantitatively identifying hotspots (Fig 6 and S1 Table with the statistical data). Although which specific traps were parts of hotspot clusters differed between high vs. low-rain seasons within years and among years within the high vs. low-rain seasons, there was repeatable clustering of traps with high *Ae*. *aegypti* captures in the upper-middle region of Captiva in all years, that is a clear hotspot where control efforts should be targeted (Fig 6). Further, another hotspot was repeatable across the final two years of monitoring to the south of the persistent mid-island hotspot on Captiva that should also receive increased operational scrutiny in future work (Fig 6).

**Fig 7:**
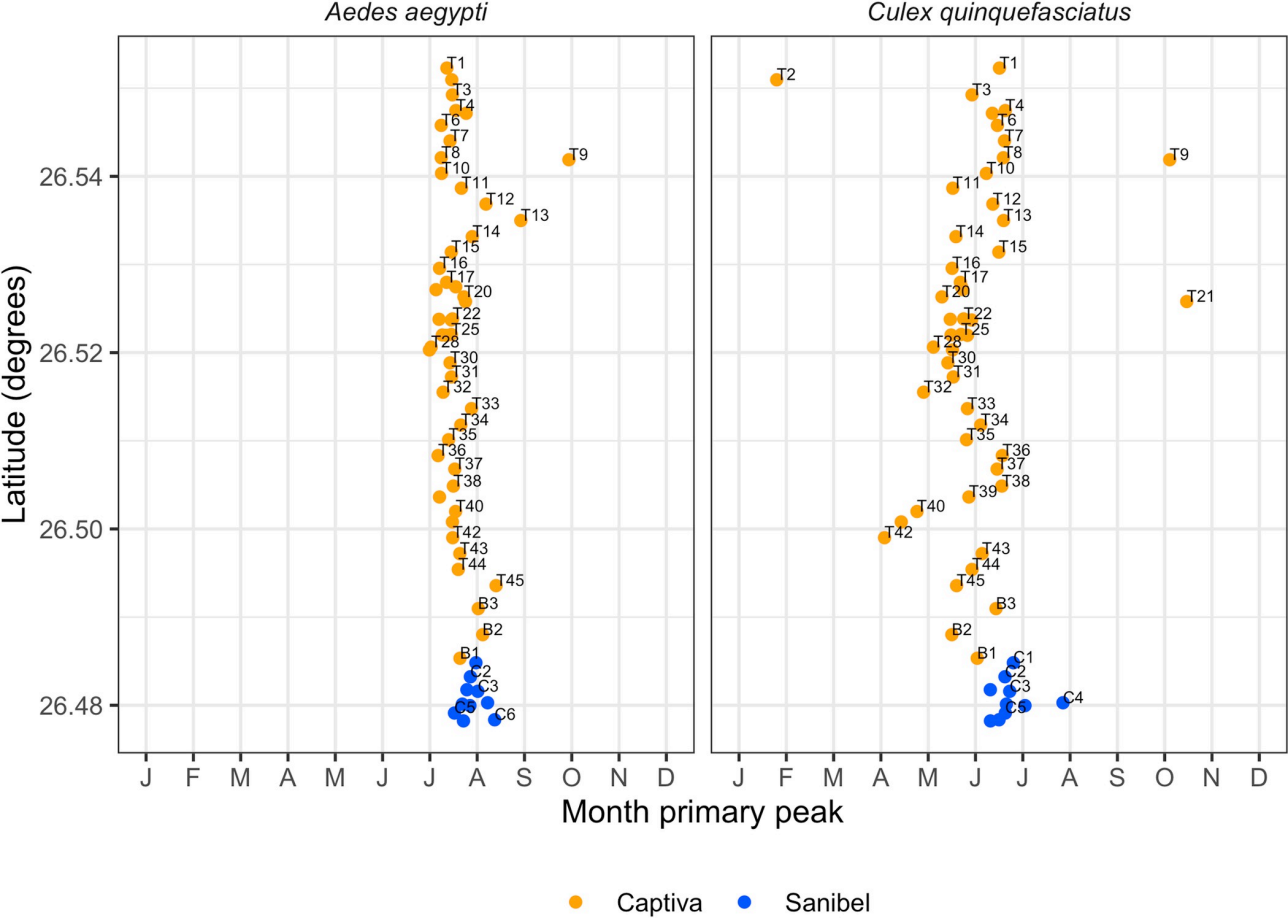
Species primary. Monthly primary peak describing the month with the highest collection peak of collection between mid-2017 to mid-2020, by trap position (latitude trap distribution) for *Ae*. *aegypti* (A) and *Cx*. *quinquefasciatus* (B).

Taking advantage of the almost linear trap distribution from north to south on Captiva and Sanibel, we visualized the seasonal peaks of peaks of *Ae*. *aegypti* and *Cx*. *quinquefasciatus* by plotting the month in which trap capture was highest in relation to trap latitudinal distribution (Fig 7). Most of the peaks for *Ae*. *aegypti* were concentrated between July and September, with just one trap (T9), having a peak towards October. On the other hand, a wider seasonal distribution over the months was observed for *Cx*. *quinquefasciatus*, with at least three traps (T2, T9, and T21) with distinctly different seasonal peaks from the majority. Notably, the peak timing for both species in T9 peaked later than most of the other traps, suggesting the area in which this trap is located may be locally cooler during the hottest summer months. Interestingly, we also note that the peak month for *Ae*. *aegypti* was more synchronized among traps than the peak month for *Cx*. *quinquefasciatus*, which was more variable across traps.

## 4. Discussion

Detailed, multi-year entomological data collection constitutes a fundamental source of information to understand the population dynamics of target species before initiating any innovative control tactic, including our proposed SIT trial for *Ae*. *aegypti*. Because mosquito populations can fluctuate from season to season and year to year at the same location, a longer timeframe of background surveillance increases understanding about the stability of mosquito populations in a target area, thus facilitating better planning and implementation of control tactics. Furthermore, longer-term data collection allows practitioners to more clearly evaluate the extent to which declines in populations at a location are likely to be due to the implementation of a control tactic versus natural seasonal or year-to-year variation in baseline population densities [21]. The baseline entomological surveillance data recorded in this study from 2017–2020 provides us with a solid understanding of the unique dynamics of the *Ae*. *aegypti* populations found in Captiva and northwest Sanibel Islands, providing a base from which we can launch an operational SIT program for Captiva Island. Furthermore, the data gathered in this study provides the appropriate timing for SIT implementation, and a more precise understanding of the numbers of irradiated males needed for releases. We can now target effective release areas increasing the potential for success in implementing and evaluating the success of *Ae*. *aegypti* population suppression in a radiation-based SIT field trial [11, 15], such as those that have recently been successfully performed on *Ae*. *aegypti* in Cuba [44] and Thailand [34] as well as for *Ae*. *albopictus* in Greece [21] Spain [22], and Albania [23], among others. Because we also have spatially and temporally explicit long-term data on other mosquito species that co-occur with *Ae*. *aegypti*, we will be able to determine whether suppression of *Ae*. *aegypti* is correlated with increases in the populations of any of these other mosquito species in the community.

Environmental factors, such as temperature and precipitation, greatly influence *Ae*. *aegypti* populations and are often responsible for the seasonality and dynamics of this species. In some cases, human behaviors in response to these factors may impact mosquito populations indirectly, i.e. construction of water storage in areas with low precipitation [35–37]. On Captiva and Sanibel, both precipitation and temperature modulate the mosquito population in such a way that the population of *Ae*. *aegypti* practically disappears from both areas during the low-rain season and lower temperature, returning when the temperature and precipitation increase next season. This pattern was also seen in a study in Brazil from 2016 to 2018, in which the environmental conditions had a significant role in defining the population profile in the study site, thereby also influencing the epidemiological profile [38]. Extreme weather drastically affected the populations of *Ae*. *aegypti* when Hurricane Irma hit Captiva and Sanibel Island in September 2017. The year 2018 should be considered a “recovery” phase given the significance of the hurricane’s impact, as populations were lower over the year following the hurricane but had a resurgence in subsequent years. Natural disasters are of particular concern due to the increased availability of breeding sites, and can result in a newly emerged population that can facilitate pathogen transmission and cause the rise of vector-borne disease incidents [20, 39, 40].

Autocidal interventions, such as SIT, should ideally start when the target population is at its lowest density. The monthly primary peak analysis, shown in Fig 6, is a potent tool because it can reveal temporal peaks for each trap that are valuable indicators of where and when interventions should begin. This analysis also reinforces the link between population density and precipitation levels because the rainy season typically starts in May and the *Ae*. *aegypti* population starts growing, with population density then peaking between July to September (throughout the studied period). The importance of weather data in predicting *Ae*. *aegypti* population levels have been previously reported, as well as the high positive correlation between density levels with precipitation and temperature [41]. The data from this study suggest that future sterile male releases may be most successful if implemented before mid to late April before the high-rain season begins and well before the peak population density typically occurs. While weather data are an invaluable tool to understand fluctuations in the *Ae*. *aegypti* population, it should be noted that the existing egg bank is another complicating factor that will also contribute to population size, particularly early in the high-rain season. The presence of persistent egg banks in *Ae*. *aegypti* further necessitates continued surveillance once a population suppression intervention begins to indicate what adjustments may be needed in the number of sterile males released and the locations of those releases to achieve the best possible results for population suppression.

Documenting the spatial distribution of *Ae*. *aegypti* in the field can also reveal the dynamics of this species in space and time. Because *Ae*. *aegypti* flourishes in peridomestic habitats, the anthropogenic structure of a particular location may create new breeding sites or attract mosquitoes from other areas to specific hotspots that can be identified with BGS traps. The hotspots identified in this study will allow us to target interventions to areas where they can be most effective in suppressing wild *Ae*. *aegypti* populations. For example, continued high-density weekly trapping efforts during sterile male releases will allow us to adjust releases to compensate for shifts in wild *Ae*. *aegypti* population densities over the course of our planned SIT intervention, including compensating for changes in human use patterns that increase biting opportunities or changes to the environment, such as new construction or changes in vegetation that may alter the distribution of breeding sites [42–44].

Of course, an outstanding question for a species-specific suppression program is whether there will be impacts on other mosquitoes in the community. Removing a dominant, competitive mosquito like *Ae*. *aegypti* may not reduce biting pressure on residents and visitors to these vacation-spot islands if another anthropophilic species simply replaces *Ae*. *aegypti* by increasing their densities. For example, historically *Ae*. *albopictus* had not been detected on Captiva or Sanibel Islands, which is one of the reasons why these sites were selected for our upcoming *Ae*. *aegypti* SIT pilot trial. During our baseline study, three individual *Ae*. *albopictus* were trapped in 2018 and 2019; one in Captiva, the release site, and two in Sanibel, the control site. As reported by Lounibos and colleagues, who studied the population dynamics of *Ae*. *aegypti* and *Ae*. *albopictus* in several cities in peninsular Florida (USA) over a 20 year period (1994–2014), the two species tend to find equilibrium after waves of displacement [45]. Whether *Ae*. *albopictus* may become established in our field sites after SIT suppresses or eliminates the *Ae*. *aegypti* population on Captiva Island remains to be seen. However, there are contradictory reports about this in the literature [46, 47]. If population replacement of *Ae*. *aegypti* by *Ae*. *albopictus* does occur after a species-specific intervention, consistently monitoring *Aedes* mosquitoes in these areas would immediately trigger a response for species-specific control of *Ae*. *albopictus*, a species for which there are several species-specific control measures, including a complementary SIT package is already available and has been successfully used in field suppression [12–15, 48, 49].

## 5. Conclusion

Establishing a baseline data collection process is fundamental for planning innovative mosquito control applications and essential to implementation. Continuous trapping is critical to determining the population profile, along with abiotic parameters to predict its fluctuations and peaks as indicators for planning actions and interventions, for example in a phased conditional approach for implementing SIT. Keeping records for additional species beyond the target of the innovative control measure allows better characterization and comparative assessment of the study areas. Importantly, substantial pre-intervention knowledge of the whole mosquito community in a site can allow assessment of whether suppressing a target species with species-specific interventions lead to compensatory increases in the abundances other potentially concerning mosquito species. The authors believe that the data collected in this study from 2017–2020 has provided a fundamental understating of the *Ae*. *aegypti* population on Captiva and northwest Sanibel Island, providing the needed baseline data to properly evaluate the effectiveness of future innovative interventions, including a SIT program planned to be implemented on these islands.

## Supporting information

S1 Fig Heatmap. Heatmap of mosquito distribution of diversity and dominance indexes during the high and low rainy seasons. Lat = latitude and Long = longitude.
(TIF)

S1 Table Data used for the analysis presented in this manuscript. (CSV)
