# Supplementary figures and images for: Seasonal insights for integrative mosquito management from multi-year baseline entomological data on Aedes aegypti in Lee County, Florida
Source: PLoS One. 2024 Oct 11;19(10):e0311407. doi: 10.1371/journal.pone.0311407 (PMC11469506; doi:10.1371/journal.pone.0311407)

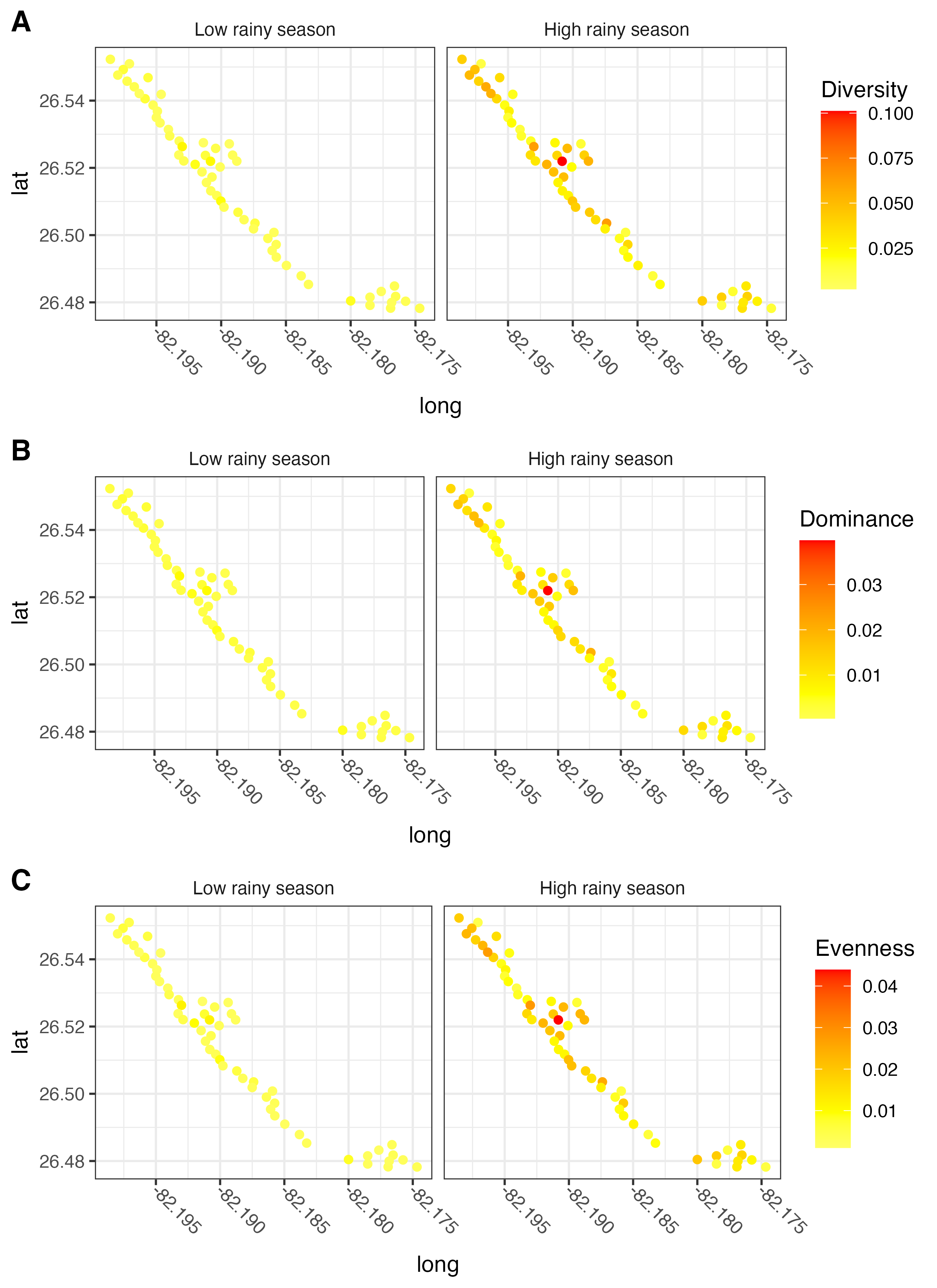

Supplement: S1 Fig — Heatmap of mosquito distribution of diversity and dominance indexes during the high and low rainy seasons. Lat = latitude and Long = longitude. (TIF) [file pone.0311407.s001.tif]
